# *Toxoplasma* on the Brain: Understanding Host-Pathogen Interactions in Chronic CNS Infection

**DOI:** 10.1155/2012/589295

**Published:** 2012-03-22

**Authors:** Sushrut Kamerkar, Paul H. Davis

**Affiliations:** ^1^Department of Biology, University of Nebraska at Omaha, Omaha, NE 68182, USA; ^2^Department of Genetics, Cell Biology & Anatomy, University of Nebraska Medical Center, Omaha, NE 68198, USA

## Abstract

*Toxoplasma gondii* is a prevalent obligate intracellular parasite which chronically infects more than a third of the world's population. Key to parasite prevalence is its ability to form chronic and nonimmunogenic bradyzoite cysts, which typically form in the brain and muscle cells of infected mammals, including humans. While acute clinical infection typically involves neurological and/or ocular damage, chronic infection has been more recently linked to behavioral changes. Establishment and maintenance of chronic infection involves a balance between the host immunity and parasite evasion of the immune response. Here, we outline the known cellular interplay between *Toxoplasma gondii* and cells of the central nervous system and review the reported effects of *Toxoplasma gondii* on behavior and neurological disease. Finally, we review new technologies which will allow us to more fully understand host-pathogen interactions.

## 1. Introduction

*Toxoplasma gondii* belongs to the phylum Apicomplexa, which consists of intracellular parasites having a characteristically polarized cell structure and a complex cytoskeletal and organellar arrangement at their apical end [1]. This obligate intracellular parasite can infect and replicate within virtually any nucleated mammalian or avian cell [2, 3]. It is believed that the major transmission method of *T. gondii* to humans is the consumption of raw or rare meat [4–6]. In addition, vertical transmission of *T. gondii* is also possible, occurring when a female receives a primary infection while pregnant which can lead to fetal morbidity such as hydrocephaly. Indeed, *T. gondii* infection is a primary cause of fetal malformations in the United States [7]. Up to 80% of a population may be infected, depending on eating habits and exposure to felines, which serve as the definitive hosts and shed environmentally robust oocysts in feces [7, 8]. Oocysts can be stable in the environment for up to a year, may contaminate food or water supplies, and infect other warm blooded vertebrates [9]. A recent study suggested that oocyst-acquired infections are the most clinically severe form of infection, which may occur not just through direct cat fecal exposure, but contamination of municipal drinking water [10].

Two critical intracellular stages in the pathogenesis and transmission of *Toxoplasma gondii* are the rapidly replicating tachyzoite stage and the slower growing, cyst-forming bradyzoite stage. Initially, latent infections in humans were assumed to be largely asymptomatic. However, during the initial AIDS crisis, *Toxoplasma* became known as a major opportunistic pathogen [11]. As the host adaptive immune response weakens, parasite tissue cysts rupture and release bradyzoites through an unknown mechanism. These recrudescent infections permit parasite conversion to the rapidly-dividing tachyzoite stage and produce significant morbidity, including *Toxoplasma* encephalitis [12, 13].

Until recently, *T. gondii* chronic infections were considered largely innocuous in the otherwise healthy patient, despite observed neurological changes. However, more recent studies on model animals have suggested that behavioral changes are manifest following infection [14]. Moreover, recent associations have been made between parasite infection and neurological disorders, such as schizophrenia [15]. Hence, it is critical that the relationship between both host and parasite, and between infection and disease, be subjected to more analysis. Central to these issues is the involvement of the host immune response, which is only beginning to be delineated and understood.

## 2. Acute Infection and Dissemination

The most frequent cause of primary infection is the ingestion of *Toxoplasma gondii* tissue cysts. Surviving the gastric processes, the parasite excysts to cross into intestinal epithelium and continues propagation [16]. Due to advantageous intracellular localization, the parasite is largely protected from soluble, humoral, or cellular antimicrobial factors, although the degree of success may be dependent on the parasite genotype [17]. However, a T_H_1 immune response is nevertheless triggered during this acute stage, as recently reviewed in [18, 19]. The parasite has developed adaptations which allow it to manipulate the innate immune system, frequently leading to continued proliferation in the gut tissue, despite the influx of lymphocytes and cells of the innate immune system [20]. Paradoxically, it is believed that these cells, particularly dendritic cells and macrophages, are intracellularly infected and grant the parasite the ability to spread hematogenously via a “Trojan horse” approach [21–23].

Once in circulation, parasites are able to migrate within infected cells and remain in the tachyzoite state prior to activation of the adaptive immune response [24]. Thereafter, parasites somehow become confined to muscle and brain tissue [25]. In a process poorly understood, the parasites are believed to traverse the endothelial cells comprising the blood brain barrier. A recent study by Lachenmaier et. al suggests that infected murine brain endothelial cells promote infected leukocyte migration through the blood brain barrier [26]. Whether other mechanisms, such as extracellular parasite barrier penetration, are used to gain access to the CNS is still unknown.

## 3. Bradyzoite Formation

The chronic, robust bradyzoite stage is critical for the transmission of the parasite via carnivorism and likely accounts for parasite ubiquity. Tissue cysts are composed of host cells which may contain 100 or more individual parasites surrounded by a cyst wall produced during differentiation. The transition to the chronic stage is thought to be induced by exogenous stressors to the parasite, host, or both or may occur spontaneously depending on infected cell type [27–30]. According to Blader and Saeij, neurons and muscle cells are terminally differentiated and withdrawn from the cell cycle. They have suggested a model in which tachyzoite growth is favored inside of growing cells, but when tachyzoites cannot manipulate the host's cell cycle, bradyzoite development initiates [31].

The most physiologically effective method of bradyzoite stage induction *in vitro* is increasing the pH of the culture media to 8.0–8.2, although variations of this method exist [32, 33]. Exposure of *Toxoplasma gondii* to an alkaline media prior to host cell invasion enhances bradyzoite differentiation [34]. Alternatively, heat shock (43°C) of the host cells for 2 hours prior to invasion followed by parasite invasion for 2 hours at 37°C and additional heat shock of infected cells for 12–48 hours after infection is an induction method less harsh to host cells [32]. Chemical induction methods, such as the use of sodium arsenite, sodium nitroprusside, or a trisubstituted pyrrole (Compound 1), are also effective [32, 35, 36]. Nutrient deprivation, such as the amino acid arginine, slows growth and enhances differentiation [27, 37]. Simultaneous inhibition of pyrimidine de novo biosynthesis and salvage pathways (via low CO_2_) also induces slow growth and differentiation to bradyzoites [38]. Alteration of host cell gene expression has been shown to slow tachyzoites replication, which may induce bradyzoite specific gene expression [39]. Thus, application of exogenous stress to the parasite appears to consistently trigger the formation of the bradyzoite state *in vitro*.

Due to the clinical importance of the bradyzoite stage, and the ability to generate this stage *in vitro*, it has been the focus of several studies [12, 27, 33, 40–42]. The *T. gondii* cyst wall membrane, largely consisting of glycoproteins, is thought to be critical in maintaining the structural and nutrient needs of the parasite while mitigating host immune system detection [43–45]. Additional observable changes occur in subcellular organelles, including a decrease in dense granules, and an increase in micronemes and large amylopectin granules. The parasite downregulates cell division and enters a quiescent G_0_ state [28], and general protein translation slows considerably due to parasite eIF2 phosphorylation [46, 47]. Interestingly, knocking out an abundant protease inhibitor in the parasite led to enhanced bradyzoite formation *in vitro* [48]. Transcriptional profiles of high-resolution timecourse experiments of tachyzoites undergoing differentiation are available at eupathdb.org [49–51]. These studies include parasite transcript measurements from multiple strains subjected to a variety of induction conditions, including CO_2_ starvation, sodium nitroprusside, alkaline media, or Compound 1 treatment. Results from these studies not only confirm the upregulation of known bradyzoite markers, but also reveal a novel set of early upregulated transcripts (Davis PH, manuscript in preparation).

According to Sullivan et al., the bradyzoite cyst form strongly contributes to the success of *Toxoplasma* in the following manner [12]: (1) the cyst survives gastrointestinal processes, allowing invasion of the small intestine; (2) the cyst is resistant to host immune response (and current drug treatments); (3) the parasites persist without perturbing host cells throughout the lifespan of the host; (4) bradyzoites in tissue cysts are infectious, lending to carnivorous transmission.

## 4. Immune Response to CNS Infection

Upon entering tissues of the central nervous system, the parasite establishes a delicate balance of low metabolic and proliferative activity, while avoiding robust host immune system activation [52]. Meanwhile, it is advantageous for the host to balance prolific replication of the pathogen with the potential for intense immunopathology. While most subclinical infections of *Toxoplasma* demonstrate this balance, it should be noted that the interplay between various host and parasite genotypes allows for considerable variation in observed immune response and course of infection [53–57]. Due to the difficulties in studying human CNS infections, most reported information concerning the immune response in *T. gondii* CNS infection originates from murine models. In recognition of known immunological differences between mice and humans, cross-species comparisons of effector molecules can be difficult [58, 59]. However, these models have yielded substantial understanding of the cellular immunoregulation of *Toxoplasma* infection [19]. Several studies of the effects of *Toxoplasma* infection on cells of the CNS have been compiled in Table 1.

Upon entry to the CNS, tachyzoite parasites appear to infect astrocytes, neurons, and microglial cells, possibly with different affinities. Parasite infiltration is followed by CD4^+^ and CD8^+^ T cell influx in a process still not fully understood, but which is critical for control of *T. gondii* CNS infection, and which can be activated via CD28 or ICOS stimulatory pathways [60–64]. Infection and subsequent lymphocyte infiltration is reported to cause structural modifications to CNS tissues, based on two-photon image observations [65]. Cellular components of the innate response, such as macrophages and NK cells, are also able to enter the CNS during infection, but their role is less clear. A main feature of influxed activated T cells is the production of IFN-gamma, shown to be essential for the prevention of parasite reactivation in an immune cell-mediated manner [66, 67]. To a lesser degree, microglial and other cells also generate IFN-gamma, as well as several other pro- and anti-inflammatory cytokines and chemokines following infection [68–72]. *In vitro* work suggests that astrocytes and microglial cells are able inhibit parasite replication upon activation [73–75], possibly explaining why neurons are the dominant chronically infected cell type [76, 77]. Moreover, the process of parasite clearance appears reliant on host cell autophagy [78, 79]. However, a recent report suggests that microglial cells may function as a “Trojan horse” in the dissemination of recrudescent parasite infection [80].

During and following acute CNS infection by *T. gondii*, the host must maintain a balance of controlling parasite proliferation, while avoiding immunity-induced damage. The inhibitory effect of IL-10 is required to prevent immunopathology during primary infection, but not required to prevent immune hyperactivity during secondary challenge to *T. gondii, *nor required to generate a memory response [81]. IL-27 has also been described as immunosuppressive in the context of toxoplasmosis and may induce IL-10 production [82–84]. Immune-related pathology is also believed to be locally controlled by inducible TIMP-1, an inhibitor of matrix metalloproteinases (MMPs) produced by astrocytes and other microglial cells [85]. Upon CNS infection by the parasite, T cells migrating into the CNS have shown increased expression of MMP-8 and MMP-10, proteins involved in tissue remodeling, cell migration, and inflammation. The absence of the MMP inhibitor TIMP-1 reduced parasite load approximately four-fold, but it is predicted that additional CNS damage would occur in the presence of untempered MMP activity [86, 87].

Once a chronic infection is established, the parasite is predominantly found in the bradyzoite stage within the CNS. Based on microscopic studies, cysts were located throughout the brain, but concentrated in the cerebral cortex, hippocampus, basal ganglia, and amygdala [88]. The cyst stage dominance may be due to at least two phenomena: first, the acute immune response may successfully clear cells infected with the tachyzoite stage, leaving only bradyzoite-containing cells to remain viable. Second, the interferon-gamma upregulation associated with the acute response may maintain parasite differentiation [27]. Recent studies have shown that, unlike extracellular parasites, cyst-bearing cells are not visible to CD8^+^ T cells, suggesting that such intracellular cyst structures are an effective means of immune evasion [89]. Alternatively, this data may be explained by the relatively low MHC class I displayed by neurons. Additionally, T cell behavior has been shown to be dependent on antigen availability in the CNS [65].

Of note, various alterations in the host immune response have been shown to allow recrudescent disease, hallmarked by parasite conversion back to tachyzoites and ultimately toxoplasmic encephalitis [90]. The clinical relevancy of this finding became apparent during the onsets of the AIDS epidemic [91]. However, in most immunocompetent conditions, parasite infections will remain in a chronic subclinical state (aside from possible behavioral modifications, discussed below) for the lifespan of the host. Whether bradyzoite cysts regularly (or randomly) burst open in immunocompetent hosts and quickly reinvade nearby cells is an unsettled question [13]. It is possible that infrequent cyst release is met with a robust memory response which eliminates some or all extracellular parasites prior to reinvasion. Or the bradyzoite cysts may simply be capable of outlasting the host. Likely, some combination of these events contributes to the long-lasting balance demonstrated by the interaction of the host and parasite, thus making it one of the most prevalent parasitic infections globally.

## 5. Exploring the Effects of *Toxoplasma gondii* on Behavior

Certain parasites have been known to selectively alter host behavior to enhance their transmission. Although latent infection with *Toxoplasma gondii* is among the most prevalent human infections, it has been assumed to be mostly asymptomatic, despite early work showing deleterious memory effects on murine models [92]. More recently, it has been found that the parasite has the ability to modify host behavior. Infected rats were shown to be less fearful of cats (the definitive host of the parasite) as compared to noninfected controls, thus conferring a sexual advantage to the parasite [93]. This has lead researchers to speculate whether the parasite may have similar effects on humans [14, 94]. It is unknown whether these behavioral changes in the host are due to the parasite alone, or are they due to the outcome of the host's immune response against the parasite. Alternatively, such effects could be side effects of host illness or even a fortuitous byproduct, such as inducing the host to undertake greater risks to meet higher energy demands [95, 96]. For example, infected rats are more active than uninfected counterparts [97]. Intriguingly, infected rats are less neophobic (fear of novelty) to each novel stimuli presented, as compared to uninfected rats [98]. While some infected rats showed a strong aversion to areas with cat odor, a proportion of infected rats showed a potentially sexual attraction to cat-treated areas [93, 99].

The behavioral manipulation hypothesis postulates that a parasite will specifically manipulate host behaviors essential for enhancing its own success [14, 100]. However, the neural circuits involved in learned fear, anxiety, and innate fear overlap to a great extent, suggesting that the parasite may disrupt all of these nonspecifically [95]. One group has reported that the density of cysts in the medial and basolateral amygdala is almost double that in other structures such as hippocampus, olfactory bulbs, and prefrontal cortex [95]. The amygdala performs a primary role in the processing of memory and emotional reactions, such as fear. This may be the reason why infected mice show a nonwildtype attraction to feline odor and/or have modified fear, or sexual arousal responses. Hence, in this context, the behavioral manipulation hypothesis would support the capacity of the parasite to ameliorate innate feline fear, and possibly replace it by a novel or feline attraction, while appearing to leave other domains unchanged [101]. To date, however, there is no known mechanism coordinating infected regions with changes in behavior.

To the degree that these can be measured, nonmemory-related cognitive functions, anxiety, and social behavior in infected mice are unchanged when compared to controls; yet, they experience profound and widespread brain pathology, motor coordination, and sensory deficits [102]. These changes could be due, in part, to hyperactive MMP proteolysis [103], and/or the creation of novel brain structures [65], as discussed above. It has been proposed that CNS modification following *T. gondii* infection may behaviorally affect human hosts, as well [96]. There have been published correlations between latent *Toxoplasma* infections and human behavioral changes such as: slower reactions, lower rule consciousness, decreased novelty seeking behavior and greater jealousy in men, and promiscuity and greater conscientiousness in women, as reviewed in [96]. *Toxoplasma gondii* can increase the dopamine levels in rodents [104]; this may be due to the inflammatory release of dopamine by increasing cytokines such as interleukin-2, or potentially by direct parasite production. Many of the neurobehavioral symptoms that are postulated to be due to toxoplasmosis correlate to the general function of dopamine in the human brain.

## 6. *Toxoplasma*-Associated Psychiatric Sequelae

The dopamine imbalance between the mesolimbic and the mesocortical regions in the brain is suspected to play a role in the development of schizophrenia. This may permit a relationship between schizophrenia and toxoplasmosis [96]. Schizophrenia is one of the most prevalent and severe psychiatric syndromes. With onset often in young adulthood, schizophrenia is characterized by impairment in thought processing, perception, cognition, mood, and psychomotor behavior [15]. There is a growing interest in the role of parasites in the causation of psychiatric disorders, in addition to personality changes, and risk-taking behavior. Of note, drugs that have antipsychotic and mood stabilizing properties (which are used in the treatment of schizophrenia and other psychiatric disorders) may be augmented through their inhibitory impact upon *T. gondii* in infected individuals [94]. An example of this is the antipsychotic haloperidol and the mood stabilizer valproic acid, which most effectively inhibit *Toxoplasma* growth *in vitro*, although not *in vivo* [105].

To date, no causal link has been demonstrated, but correlative data is abundant. For example, 185 noninebriated automobile drivers in Turkey involved in a vehicular accident within a 6-month window were evaluated for toxoplasmosis. The cohort of drivers involved in accidents was substantially more likely to have *T. gondii* infection compared to the control (nonaccident) group: 33% versus 8.6% seropositive, respectively [106]. A number of studies have assessed seropositivity to *Toxoplasma gondii* in individuals with schizophrenia and other forms of severe psychiatric disorders, with inconsistent correlative results [107–109]. In addition, *Toxoplasma gondii* encephalitis may manifest with symptoms similar to those of schizophrenia and other psychiatric disorders [110]. There have been a high number of cases with symptoms that included delusions, thought disorder, and auditory hallucinations in patients with AIDS and toxoplasmic encephalitis [15, 110].

*Toxoplasma gondii* infection has also been associated with obsessive-compulsive disorder in humans [15]. Men had “lower superego strengths (rule consciousness) and higher vigilance” as well as being “more expedient, suspicious and jealous.” These factors are associated with substance abuse, anxiety, and personality disorders. Women showed almost the opposite behavior: with higher superego strength and factors that suggested warmth, conscientiousness, and moral adherence. But both men and women were found to have more apprehension compared with uninfected controls [15, 96]. According to Flegr, differences in the level of testosterone may be another reason for these observed differences [96]. High testosterone individuals may be more susceptible to *Toxoplasma* infection via a less robust immune response, or observed behavioral changes could be the result of the parasite inducing testosterone availability in order to further impair the cellular immunity of the host. In a small study, seropositive men were found to have higher concentrations of testosterone than uninfected men; however, it is unknown whether high testosterone predisposes individuals to infection behaviorally or biologically, or whether the parasite indirectly drives testosterone levels. In an ongoing high-throughput cell-based screening study, overexpression of 17*α*-hydroxylase in human cells substantially increased the *in vitro* rate of *Toxoplasma* growth, while the inhibition of this transcript via siRNA decreased intracellular growth (Davis PH, manuscript in preparation). 17*α*-hydroxylase is a key metabolic enzyme responsible for converting cholesterol-like molecules into androgen precursors, such as testosterone. This finding suggests that testosterone-like sterols may directly benefit the growth of the parasite.

## 7. Future Directions

Due to the growing possibility that *T. gondii* infection can alter host behavior, there may be a renewed push for antiparasitic agents, as chronic* Toxoplasma gondii* is untreatable. Agent development may be difficult, however, due to the need for drugs to penetrate the blood-brain barrier, as well as the parasite cyst wall [111]. Moreover, even if the parasites could be removed from the neurons without creating additional tissue destruction, preexisting tissue pathology may preclude resolution of possible behavior-related sequelae. Recently, a study identified several compounds capable of inhibiting *T. gondii* tachyzoites *in vitro*, in addition to *P. falciparum* [112], and some of these compounds are being investigated for their antibradyzoite properties (Davis PH, manuscript in preparation).

In addition, the growing understanding of the complex immunoregulatory processes surrounding parasite infection may aid possible vaccine development [113]. However, Table 1 indicates the paucity of information on the interplay between the immune system and the bradyzoite stage, which may be a valuable avenue for future exploration. Future work may also be directed at delineating the process of parasite penetration through the blood brain barrier, as well as a deeper understanding of the molecular events in T cell control of infection. Much like the contributions of electron microscopy illuminated our understanding of apicomplexan organisms [114], so too does advanced imaging, such as bioluminescence and two-photon imaging, promise to provide greater details and real-time information on the workings of this parasite and its interactions with the host [65, 89, 115–119]. Moreover, the precise role of antigens and host immune cells promises to be robustly detailed with tetramer-based molecular tools [61]. Finally, host modification, such that siRNA and overexpression of host genes, may illuminate critical cellular factors required for the parasite's lifecycle [120]. Hi-throughput cell-based screening promises to hasten this understanding considerably.

## Figures and Tables

**Table 1:** The response of CNS-resident cells to *Toxoplasma gondii *infection.

Brain cell type	Parasite stage	Activity	Reference
Neuron	Tachyzoite	Parasites can encyst in neurons	[75]
Neuron	Tachyzoite	Infection induces cytokine and chemokine production; stimulated neurons are unable to inhibit parasite growth	[121]
Neuron	Bradyzoite	Neurons containing parasite cysts avoid scrutiny by CD8^+^ T cells	[89]
Neuron, microglia	Tachyzoite	Murine Nramp1^−/−^ models are affected in stress response and mortality following *Toxoplasma gondii* infection	[122]
Microglia	Tachyzoite, bradyzoite	Microglial cells are preferentially infected, but most effectively inhibit parasitic growth within CNS cells	[25]
Microglia	Tachyzoite	Upon *Toxoplasma* infection, microglia produce IL-1 beta, IL-10, and tumor necrosis factor-alpha	[123]
Microglia, endothelium	Tachyzoite	Murine model infection induce an upregulation of CD200R & CD200, which control CNS inflammation	[124]
Microglia, astrocyte	Tachyzoite	Infection downregulates MHC class II expression	[125]
Microglia	Tachyzoite	Toxoplasmic encephalitis induces IL-12p40, iNOS, IL-1beta, TNF-alpha largely due to CD8^+^ T cell interaction. MHC classes I and II, ICAM-1, and leukocyte function-associated antigen-1 are also upregulated	[126]
Endothelium	Tachyzoite	Toxoplasmic encephalitis induces vascular cell adhesion molecule, ICAM-1, and MHC classes I and II. Induction depends on IFN-gamma receptor	[127]
Endothelium	Tachyzoite	Infection induces ICAM-1, IL-6, and MCP-1 Induction levels vary depending on parasite strain	[26]
Astrocyte, neuron	Tachyzoite	Astrocytes are preferentially infected compared to neurons	[75]
Astrocyte, microglia	Tachyzoite	Intracellular infection reduces expressed MHC II	[125]
Astrocyte	Tachyzoite	Interferon-gamma-activated indoleamine 2,3-dioxygenase (IDO) induction inhibits parasite growth	[128]
Astrocyte	Tachyzoite	IFN- gamma induced parasite growth inhibition is independent on reactive oxygen intermediates	[129]
Astrocyte	Tachyzoite, bradyzoite	Tissue Inhibitor of Metalloproteinases-1 (TIMP-1) is induced by infection	[85]
Astrocyte	Tachyzoite	Autophagy may be involved in the elimination of the degraded parasite material from the astrocyte host cell cytoplasm	[79]
Astrocyte	Tachyzoite	IGTP is required for IFN-gamma-induced inhibition of parasite growth	[130]
